# Misdiagnosis and therapeutic impasse in psychiatry

**DOI:** 10.1192/j.eurpsy.2022.1833

**Published:** 2022-09-01

**Authors:** M. Zrelli, E. Bergaoui, N. Staali, M. Moalla, W. Melki

**Affiliations:** 1 Razi Hospital, Psychiatry D, Manouba, Tunisia; 2 Razi Hospital, Psychiatry D, Tunis, Tunisia

**Keywords:** factors of misdiagnosis, therapeutic impasse

## Abstract

**Introduction:**

We frequently receive patients with atypical psychiatric symptoms admitted in our department after consulting other psychiatrists and triying several treatments.

**Objectives:**

To highlight the factors of misdiagnosis in patients of our department.

**Methods:**

We recruited 70 patients during their appointment or during their hospital admission in our department between March and April 2021. We collected the patients’ socio-demographic and clinical data using a pre-designed questionnaire.

**Results:**

Patients were aged between 17 and 68 years with a sex ratio (M/F) of 1. Mood disorders accounted for 24.6% of disorders (N=17) whereas schizophrenia 66.7% (N=46). Patients resided in urban areas in 88.6% of cases (N=69). The average number of hospitalizations was 2.7 with extremes ranging from 0 to 14. The average time between the onset of the symptoms and the first consultation was 1 year. The mean time from onset to hospitalization was 4.37 years. The rate of consulting a psychiatrist prior to admission was 42.8%. The diagnosis was corrected during the follow-up of the patients in 24.3% of cases. Conventional neuroleptics were prescribed as first-line treatment in 42.85% of cases. Due to poor tolerance or ineffectiveness of the treatment, 31.42% of patients had to change treatment.

**Conclusions:**

Patients, who were desperate to find an adequate treatment for their disorders, put a lot of hope in the Razi psychiatric hospital. But after several years of evolution of their disease, we are faced with a therapeutic impasse. Raising awareness of mental illnesses is necessary for an early and adequate treatment.

**Disclosure:**

No significant relationships.

